# The effect of different exercise modalities on the maximal left ventricular outflow tract gradient in patients with obstructive hypertrophic cardiomyopathy

**DOI:** 10.1186/s44156-026-00141-3

**Published:** 2026-10-12

**Authors:** Maximilian Nicolas Möbius-Winkler, Andreas Hagendorff, Stephan Stöbe, Michael Gaier, Alexander Kogel, Marios Antoniadis, Ulrich Laufs, Michael Metze, Karsten Lenk, Joscha Kandels

**Affiliations:** Klinik und Poliklinik für Kardiologie, Universitätsklinikum Leipzig, Liebigstraße 20, Haus 4, 04103 Leipzig, Germany

**Keywords:** Hypertrophic cardiomyopathy, Transthoracic echocardiography, Stress echocardiography, Left ventricular outflow tract gradient

## Abstract

**Background:**

Assessment of maximally provoked LVOT obstruction (LVOTO) guides treatment decisions in obstructive hypertrophic cardiomyopathy (HCM). Guidelines recommend various maneuvers, with exercise stress echocardiography (ESE) as the preferred method if others fail. Comparative data are limited. Therefore, in this prospective study, 20 patients with obstructive HCM underwent transthoracic echocardiography (TTE) with continuous wave Doppler measuring LVOT gradients at rest, during the Valsalva maneuver, handgrip exercise at 30% and 50% of maximal hand force, squats, and bicycle ESE, with the aim of comparing commonly used maneuvers and evaluating handgrip exercise as a potential alternative for eliciting LVOTO.

**Results:**

The mean resting LVOT gradient was 38.9 ± 31.4 mmHg. Handgrip exercise caused no significant change of LVOT gradients. In contrast, gradients increased markedly during the Valsalva maneuver (98.9 ± 52.8 mmHg), squats (120.3 ± 68.8 mmHg), and bicycle ESE (83.4 ± 43.3 mmHg; *p* < 0.001 vs. rest). There was no significant difference between Valsalva, squats, and ESE (*p* = NS). Squats induced the maximal gradient in 50% of patients. Performance time was significantly shorter for the Valsalva maneuver (15 s) and squats (1 min 23 s ± 28 s) than for bicycle ESE (15 min 54 s ± 5 min 46 s; *p* < 0.01).

**Conclusion:**

The Valsalva maneuver, squats, and bicycle ESE are equally effective in provoking LVOTO, whereas handgrip exercise is not. Given their simplicity, low cost, and shorter testing time, squats may serve as a practical adjunct to the Valsalva maneuver for LVOT gradient provocation in routine clinical assessment of obstructive HCM.

**Clinical trial registration:**

German Clinical Trials Register (ID: DRKS00034473).

**Graphical Abstract:**

**Figure:**
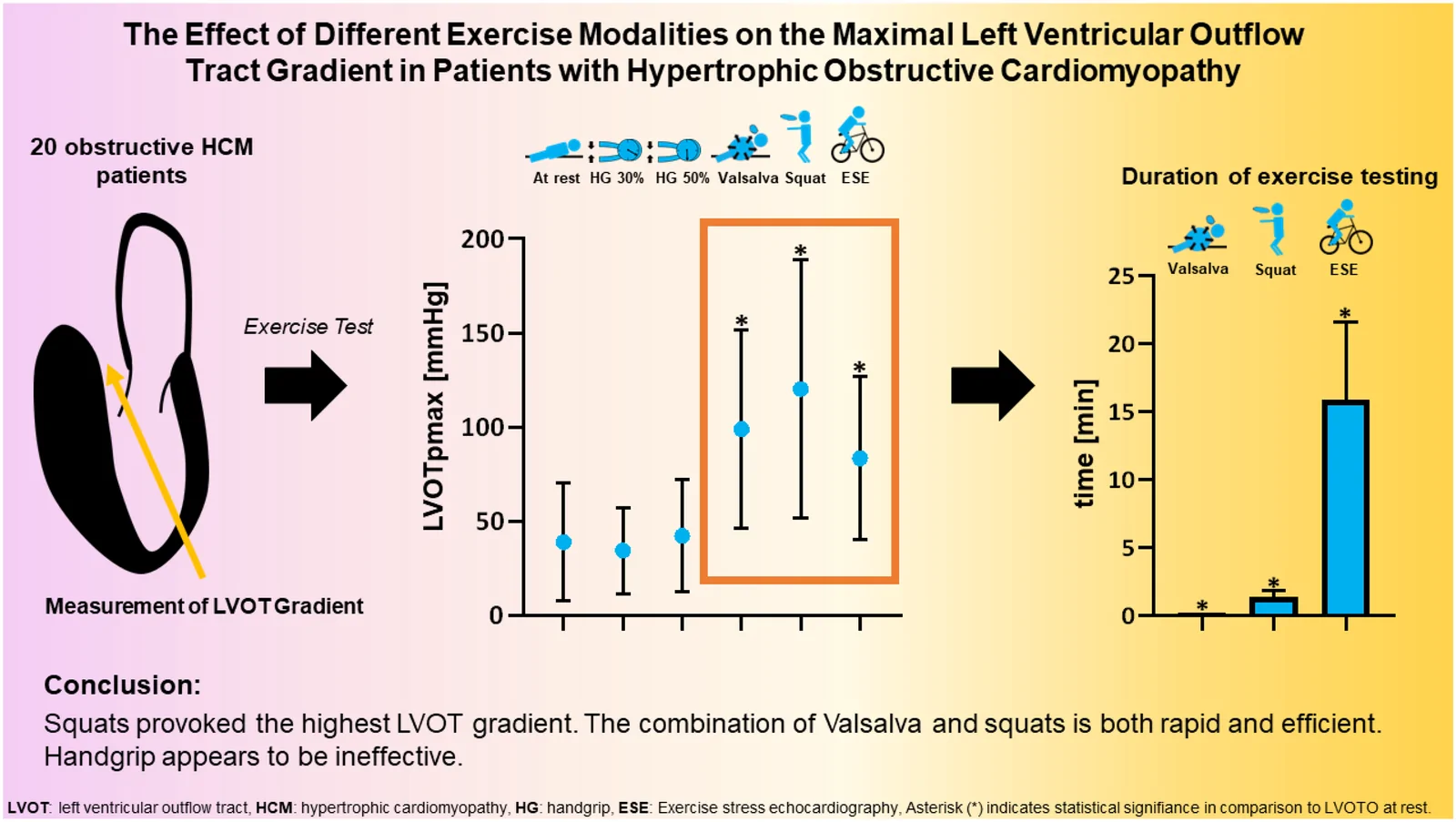

**Supplementary Information:**

The online version contains supplementary material available at https://doi.org/10.1186/s44156-026-00141-3.

## Introduction

Hypertrophic cardiomyopathy (HCM) is the most prevalent genetic heart muscle disease with a prevalence ranging from 1:200 to 1:500 [1]. Genetic transmission is predominantly autosomal-dominant, and penetrance frequently incomplete. The disease is defined by a left ventricular hypertrophy anywhere in the left ventricle ≥ 15 mm that cannot be attributed to other underlying diseases such as hypertension, aortic valve stenosis or storage disease [2]. In 70% of the patients with HCM, the left ventricular outflow tract (LVOT) is obstructed, with maximal LVOT peak gradients of ≥ 30 mmHg measured using two-dimensional (2D) Doppler transthoracic echocardiography (TTE), thus defining obstructive HCM [3].

The most frequently reported symptoms by patients with obstructive HCM are fatigue, followed by dyspnea, angina pectoris, palpitations and syncope. Notably, 34–47% of patients are asymptomatic at the time of diagnosis [4]. In addition to the symptoms, obstructive HCM is also associated with heart failure, atrial fibrillation and sudden cardiac death, regardless of the patients’ age [5, 6].

Two-dimensional TTE is usually the primary imaging modality for diagnosing HCM. LVOT obstruction (LVOTO) is a dynamic phenomenon, depending on pre- and afterload conditions. The LVOT gradient should be measured at rest and during provocation. Several bedside maneuvers are recommended in current guidelines to provoke LVOTO, particularly the Valsalva maneuver and squats [1]. In clinical practice, the Valsalva maneuver is most frequently used as it is easy to perform, rapid and inexpensive. However, it remains unclear which bedside provocation test is most effective in provoking LVOTO. Current European Society of Cardiology (ESC) and American Heart Association (AHA) guidelines recommend exercise stress echocardiography (ESE) when bedside maneuvers fail to induce a gradient ≥ 50 mmHg in symptomatic patients despite ESE being relatively time-consuming, costly and technically demanding. In symptomatic patients with maximal LVOT gradient ≥ 50 mmHg symptoms are likely attributable to LVOTO. Consequently, a gradient reducing therapy is indicated [1, 2].

Recently, handgrip exercise has been introduced as a bedside tool to assess the relevance of mitral regurgitation and led to a significant increase in MR severity grade in 29% of patients [7]. Handgrip represents an isometric form of exercise, primarily increasing afterload without significantly augmenting preload or myocardial contractility [7]. Therefore, it is not expected to have a significant impact on LVOTO. However, despite its increasing use as a simple and readily available bedside maneuver, handgrip exercise has not yet been systematically evaluated with regard to its ability to induce LVOTO in patients with obstructive HCM.

The aim of this study was therefore to compare the effects of the most common provocative maneuvers and standardized handgrip exercise on LVOT gradients in obstructive HCM patients.

## Methods

### Study population

Consecutive outpatients with obstructive HCM, who were either previously treated at or referred to the specialized HCM outpatient clinic of the Department of Cardiology of the University Hospital Leipzig were enrolled between October 2024 and September 2025. Patients were diagnosed in accordance with recent AHA and ESC guidelines by a trained, board-certified cardiologist. All patients were ambulatory, not hospitalized, had no mobility-limiting conditions that restricted their ability to perform ESE or squats and underwent TTE prior to selective myosin inhibitor therapy. Written informed consent was obtained from all participants. The study was approved by the ethics committee of the University of Leipzig, medical school (157/24-ek) and conducted in accordance with the Declaration of Helsinki.

### 2D-Transthoracic echocardiography

Two-dimensional TTE with measurement of maximal LVOT gradient at rest, during Valsalva maneuver, repetitive squats, handgrip, and bicycle ESE was performed in the departmental echo laboratory by board-certified cardiologists in accordance with the most recent guidelines of the American society of Echocardiography [8] and German Cardiac Society [9]. TTE was performed using an E95 ultrasound system with a 4Vc phased array probe (GE Healthcare Vingmed Ultrasound AS, Horten, Norway). Data were analyzed using EchoPAC software (Version 203, GE Healthcare Vingmed Ultrasound AS, Horten, Norway).

LVOTO was assessed using a continuous wave (CW)-Doppler in the apical three-chamber view (A3C) in accordance with current recommendations and our echocardiography laboratory standards [10]. Doppler beam aligned to the LVOT [11]. To evaluate maximally provoked LVOTO under different exercise modalities, patients underwent a standardized examination protocol, shown in Supplementary Fig. 1, while positioned in the left lateral supine position. All examinations were performed at 07:30 a.m. according to the examination protocol in the same way for all patients. In consideration of the recent findings that identified food intake to increase LVOT-gradients, all patients were instructed to fast for 3 h prior to the examination to ensure standardization [12]. At first, resting LVOT gradients were measured, followed by the Valsalva maneuver, handgrip exercise with 50% and 30% of maximal hand force, squats and bicycle ESE. Between every exercise modality, patients rested until full clinical recovery was achieved. This was reached when the initial heart rate and blood pressure were restored, which took an average of 5 min. Detailed descriptions of each provocation maneuver are provided below. Notably, the predefined protocol included all provocation maneuvers for all patients, regardless of the LVOT gradient measured at rest or during the preceding maneuvers. This approach was chosen to allow for a systematic, intra-individual comparison of how different provocation maneuvers affect the maximal LVOT gradient. From a clinical perspective, an LVOT gradient ≥ 50 mmHg guides treatment decisions. Once provoked, further provocation is generally not required.

### Valsalva maneuver

Patients were in a semi-supine position and were instructed to force exhalation against closed airway for 10–15 s while measuring LVOTO. This is according to the current guideline [13]. The duration of the Valsalva maneuver was calculated using a conservative approach, with a 15-second duration assumed for each patient.

### Squat-to-stand-exercise

All patients performed squats according to a standardized protocol. Squats were defined as a descending movement from a standing position with outstretched arms until a knee flexion of 90° was reached. The squats were performed at an approximated pace of one squat per three seconds (0.33 Hz). The number of repetitions was counted aloud. Patients were actively encouraged to continue until maximal subjective exhaustion. Patients immediately assumed a semi-supine position after completing the final squat. LVOT gradients were measured within 30–60 s to measure LVOT gradients in the early post-exercise phase. The duration required to perform squats was determined by recording the number of squats performed until maximal subjective exhaustion was reached. With each squat taking approximately 3 s, the total duration for each patient was calculated.

### Bicycle exercise stress echocardiography

Bicycle ESE was performed on a semi-recumbent ergometer (GE eBike, GE Healthcare GmbH, Solingen, Germany) at a constant speed of 60–70 revolutions per minute (rpm), with a semi-supine angle of approximately 45°. The test began at a workload of 50 W, increasing by 25 W every 3 min until volitional exhaustion. Each subject continued exercise for an additional 3 min recovery period at a workload of 25 W. Blood pressure, electrocardiogram and heart rate (GE Cardiosoft, GE Healthcare GmbH, Solingen, Germany) were continuously monitored throughout the examination. The standardized examination protocol is illustrated in Supplementary Fig. 2. LVOT-Gradients were measured throughout the whole examination. LVOT gradients were assessed throughout the entire examination. Measurements obtained during the early-recovery period, within 30 s after cessation of exercise, were considered the highest LVOT gradients and used for analysis. To determine the duration of bicycle ESE, the maximal workload achieved by each patient was documented. Workload increased by 25 W every 3 min, followed by a 3 min recovery period. Total exercise duration for each patient was subsequently calculated based on these parameters.

### Handgrip exercise

Patients performed handgrip exercises in accordance with a standardized protocol. A handgrip dynamometer (SAEHAN^®^ Hydraulic Hand Dynamometer; item number SH5001; SAEHAN Corp.; 23–29 Bongamgongdan 13-gil, Masanhoewon-gu, Changwon-si, Gyeongsangnam-do, 51342, South Korea) was placed in the patient´s dominant hand while in a semi-supine position at rest. The maximal voluntary hand force was measured by instructing the patient to squeeze the dynamometer with maximal effort for a few seconds. Subsequently, the handgrip exercise was performed twice, with 50% and 30% of maximal possible hand force for 3 min with 5 min rest in between. LVOT gradients were continuously recorded [7]. The procedure was overseen by a nurse, who ensured compliance with the prescribed hand force and prevented inadvertent Valsalva maneuvers during the whole handgrip exercise.

### Statistical analysis

All statistical analyses were performed using GraphPad Prism 8 and Microsoft Excel (Version Professional Plus 2016). Numerical variables were expressed as mean value ± standard deviation (SD). The assessment of normality was conducted through visual examination and the implementation of Shapiro-Wilk test. Since gradients were non-normal, differences in LVOT gradients and durations of the provocation maneuvers were assessed performing Friedman´s test and as follow-up test Dunn´s multiple comparison test was carried out. LVOT gradients at peak exercise and during early recovery following bicycle ESE were compared using the Wilcoxon matched-pairs signed-rank test. Statistical significance was assumed for *p* < 0.05.

## Results

### Patient characteristics

In total 20 patients were enrolled in this study. Baseline characteristics are depicted in Table 1. The basic echocardiographic measurements of all patients at rest are shown in Table 2.

**Table 1:** Baseline characteristics of the patients including medical background

	mean ± SD
**Patient characteristics**	
Age [years]	61.5 ± 15.5
BMI [kg/m²]	28.0 ± 5.0
BSA [m²]	2.0 ± 0.2
Male Sex (n [%])	10 (50%)
Heart rate at rest [bpm]	68.3 ± 9.5
Systolic blood pressure at rest [bpm]	144.0 ± 21.9
Diastolic blood pressure at rest [bpm]	81.1 ± 13.4
	*n (%)*
**Medical background**	
Family history of obstructive HCM	2 (10)
Atrial fibrillation/flutter	3 (15)
Arterial hypertension	11 (55)
Coronary artery disease	3 (15)
Diabetes mellitus	4 (20)
COPD	1 (5)
Asthma	0 (0)
Betablocker	14 (70)
Calcium channel blocker	6 (30)
Myosin inhibitor	0 (0)
Genetic testing performed	4 (20)
SRT	0 (0)
ICD	3 (15)
NYHA-class	
NYHA I	6 (30)
NYHA II	6 (30)
NYHA III	8 (40)
NYHA IV	0 (0)
NTproBNP levels [pg/ml ± SD]	1047 ± 1257
SAM	7 (35)
LVOT gradient < 30 mmHg at rest	9 (45)

BMI: body mass index, BSA: body surface area, SRT: septal reduction therapy, ICD: implantable cardioverter-defibrillator. obstructive HCM: obstructive hypertrophic cardiomyopathy. NYHA: New York Heart Association. COPD: chronic obstructive pulmonary disease. LVOT: left ventricular outflow tract. SAM: systolic anterior movement

**Table 2:** Basic echocardiographic measurements

Echocardiographic parameters	mean ± SD
Maximal LVOT gradients [mmHg]	
at rest	38.9 ± 31.4
Valsalva maneuver	98.9 ± 52.8
handgrip 30% of max. force	34.4 ± 22.8
handgrip 50% of max. force	42.3 ± 30.0
squat	120.3 ± 68.8
bicycle ESE	83.4 ± 43.3
IVSd [mm]	17.0 ± 3.1
LVPWD [mm]	13.2 ± 2.0
RWT	0.6 ± 0.1
LVdMi [g/m²]	150.5 ± 33.9
LVEDD [mm]	45.1 ± 5.9
LVESD [mm]	26.5 ± 5.9
LVEDVi [ml/m²]	67.2 ± 14.0
LVESVi [ml/m²]	23.4 ± 8.0
LVSVi [ml/m²]	43.8 ± 8.0
LVEF (Simpson biplane, [%])	65.8 ± 6.5
E/A	1.1 ± 0.3
E/e’	11.2 ± 3.3
LAVSi [ml/m²]	41.2 ± 11.9
TRVmax [m/s]	2.5 ± 0.3
sPAP [mmHg]	25.5 ± 6.6

LVOT: left ventricular outflow tract, IVSd: interventricular septal thickness in diastole, LVPWD: left ventricular posterior wall thickness in diastole, RWT: relative wall thickness, LVdMi: left ventricular mass indexed, LVEDD: left ventricular end-diastolic diameter, LVESD: left ventricular end-systolic diameter, LVEDVi: left ventricular end-diastolic volume indexed, LVESVi: left ventricular end-systolic volume indexed, LVSVi: left ventricular stroke volume indexed, LVEF (Simpson biplane): left ventricular ejection fraction by Simpson biplane method, LAVSi: left atrial volume indexed, TRVmax: maximum tricuspid regurgitation velocity, sPAP: systolic pulmonary artery pressure, ESE: exercise stress echocardiography

### Maximal left ventricular outflow tract (LVOT) gradients under different exercise modalities

**Fig. 1:**
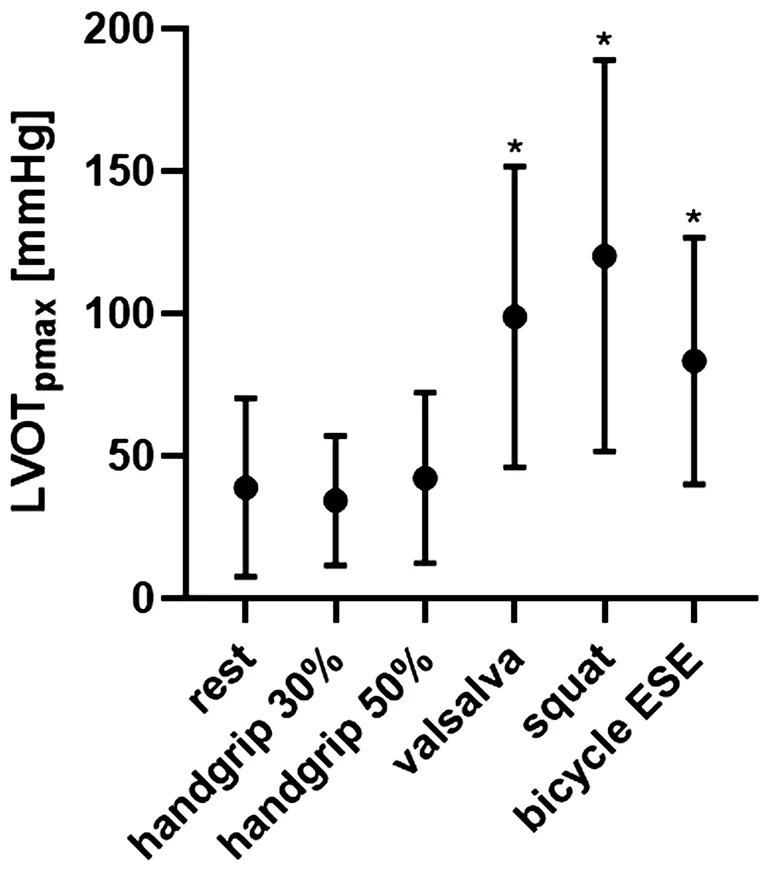
Maximally provoked LVOT gradients at rest and during different exercise modalities. Markers represent means ± standard deviation. Asterisk (*) indicates statistical significance (*p* < 0.05) in comparison with LVOT_pmax_ at rest. ESE: exercise stress echocardiography

**Fig. 2:**
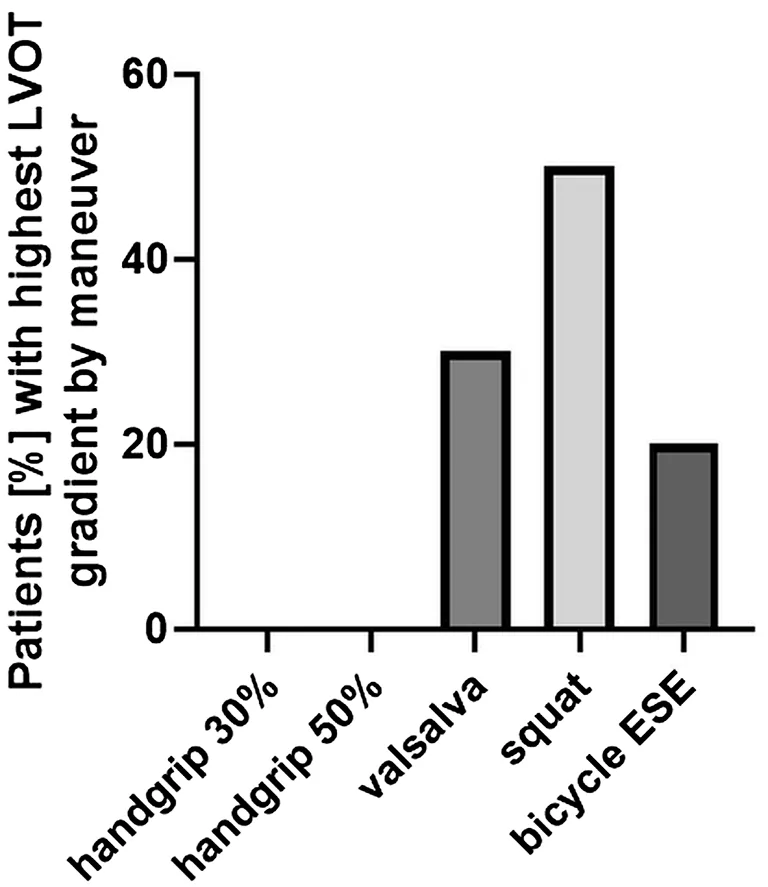
Percentage of patients with highest LVOT gradient by single provocation maneuver. ESE: exercise stress echocardiography

Patients mean resting LVOT gradient was 38.85 ± 31.35 mmHg indicating LVOTO at rest. As anticipated, handgrip exercise at 30% of maximal hand force (34.35 ± 22.81 mmHg, *p* > 0.05) and at 50% of maximal hand force (42.30 ± 29.96 mmHg, *p* > 0.05) did not significantly affect LVOT gradients. In contrast, LVOT gradients increased with the Valsalva maneuver (98.90 ± 52.81 mmHg, *p* < 0.001), squats (120.30 ± 68.75 mmHg, *p* < 0.001) and bicycle ESE (83.40 ± 43.29 mmHg, *p* < 0.001) compared to rest (Fig. 1). Of note, LVOT gradients during bicycle ESE were recorded at peak exercise and during early recovery. The LVOT gradient during early recovery was significantly higher than that at peak exercise (83.40 ± 43.29 vs. 78.26 ± 38.19 mmHg, *p* = 0.03). In 18 of 20 patients (90%), the early-recovery LVOT gradient exceeded the peak-exercise gradient. No significant difference was observed between maximal LVOT gradients provoked by bicycle ESE and either the Valsalva maneuver (*p* > 0.05) or squats (*p* > 0.05), nor Valsalva maneuver and squats (*p* > 0.05). Bicycle ESE provoked the highest maximal LVOT gradient in 4 of 20 (20%) patients, Valsalva in 6 of 20 (30%) and squats in 10 of 20 (50%) (Fig. 2). Notably, 9 of 20 patients (45%) had a resting LVOT gradient < 30 mmHg. The proportions of these patients reaching LVOT gradients ≥ 30 and ≥ 50 mmHg during the different provocation maneuvers are shown in Table 3. Within the stepwise provocation protocol, an LVOT gradient ≥ 30 mmHg was first identified during the Valsalva maneuver in 8 of 9 patients (89%); in the remaining patient (11%), this threshold was first reached during the squat maneuver. Likewise, an LVOT gradient ≥ 50 mmHg was first elicited by the Valsalva maneuver in 6 patients (67%) and was first reached during the squat maneuver in an additional 2 patients (22%). In 1 patient (11%), an LVOT gradient ≥ 50 mmHg could not be provoked by any of the investigated modalities.

Importantly, 3 of the 20 patients (15%) had an LVOT gradient < 50 mmHg during Valsalva maneuver. Of these, 2 subsequently developed an LVOT gradient ≥ 50 mmHg during both squat and bicycle ESE, whereas the other did not reach the ≥ 50 mmHg threshold during either provocation.

**Table 3:** Proportion of patients with a resting LVOT gradient < 30 mmHg reaching LVOT gradients ≥ 30 and ≥ 50 mmHg by provocation maneuver

Maneuver	LVOT gradient ≥ 30 mmHg	LVOT gradient ≥ 50 mmHg
	*n* (%)	*n* (%)
Valsalva maneuver	8 (89)	6 (67%)
Handgrip 30% of max. force	1 (11)	1(11)
Handgrip 50% of max. force	2 (22)	2 (22)
Squat	9 (100)	8 (89)
Bicycle ESE	7 (78)	6 (67)

ESE: exercise stress echocardiography

### Squats and the Valsalva maneuver are timesaving and resource-efficient in comparison to bicycle ESE

The average duration of bicycle ESE was 15 min and 54 s ± 5 min and 46 s (range 9.00–27.00 min) representing pure examination time without accounting for setup or dismantling of the bicycle ESE equipment. Patients achieved a maximal workload of 107.50 ± 48.05 W (range 50–200 W).

**Fig. 3:**
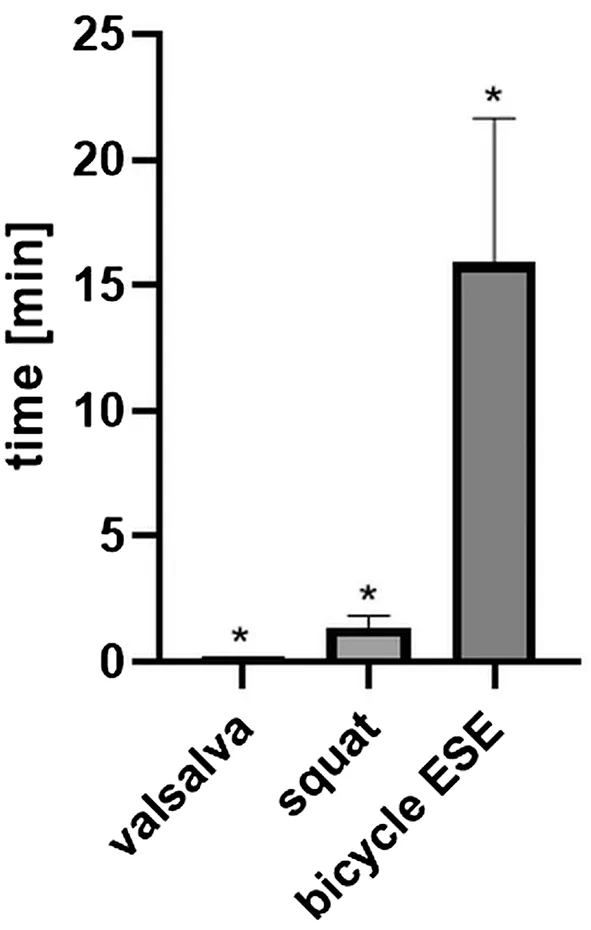
Duration of the three effective provocation maneuvers shown in minutes as mean ± standard deviation. Asterisk (*) indicates statistical significance (*p* < 0.05) for this modality when compared individually with each of the other exercise modalities. ESE: exercise stress echocardiography

In contrast, squats required 1 min and 23 s ± 28 s (range 18 s–2 min and 15 s), which was significantly shorter than bicycle ESE (*p* < 0.01). On average, patients performed 27.50 ± 9.32 squats (range 6.00–45.00 squats). The Valsalva maneuver lasted between 10 and 15 s. Using a conservative estimate of 15 s per patient, it was the shortest provocation maneuver compared to squats and bicycle ESE (*p* < 0.01) (Fig. 3). Handgrip exercise was not included in this comparison, as it appeared to be ineffective in provoking LVOT obstruction.

## Discussion

This study systematically evaluated the effects of different exercise modalities on maximal LVOT gradients in myosin inhibitor-naive patients with obstructive HCM. The primary findings of the study are as follows:

1. The mean provoked LVOT gradients did not differ significantly between squats, bicycle ESE and the Valsalva maneuver.
2. Bicycle ESE did not elicit significantly higher LVOT gradients than the Valsalva maneuver or squats, but required a substantially longer duration of the examination.
3. In the majority of patients, squats provoked the highest maximal LVOT gradient.
4. Handgrip exercise had no significant effect on LVOT gradients.

### There were no significant differences in mean provoked LVOT gradients between squats, bicycle ESE and the Valsalva maneuver

The Valsalva maneuver, squats, and bicycle ESE all significantly increased LVOT gradients compared with rest. Interestingly, there was no significant difference in maximally provoked LVOTO between these exercise modalities. Yet, it should be noted that the timing of LVOT gradient assessment differed between the modalities. While the LVOT gradient during Valsalva was measured during the maneuver, gradients following squats and bicycle ESE were assessed during early recovery. Therefore, these gradients should be interpreted as reflecting both exercise-induced and early-recovery-induced LVOTO. Notably, during bicycle ESE, LVOT gradients assessed during early recovery were significantly higher than those measured at peak exercise. This finding suggests that assessment during early recovery may capture greater LVOT obstruction than assessment at peak exercise alone and is consistent with a study by *Kim et al.* [14].

### Bicycle ESE does not provoke significantly higher LVOT gradients than the Valsalva maneuver or squats

The results of this study are in accordance with the study of *Jensen et al.*, which examined maximally provoked LVOT gradients in 57 obstructive HCM patients while performing Valsalva maneuver and treadmill ESE in a semi-supine position. The provoked LVOTO through Valsalva maneuver and ESE did not differ significantly. This study examined patients post percutaneous transluminal septal myocardial ablation during long-term follow-up [15]. In another study, *Joshi et al.* observed that both Valsalva maneuver and treadmill ESE led to an increase of maximal LVOT gradients, whereby treadmill ESE (mean LVOT_pmax_ 100 mmHg, range 0–256 mmHg) provoked significantly higher LVOT gradients compared to the Valsalva maneuver (mean LVOT_pmax_ 64 mmHg, range 0–256 mmHg). Interestingly, LVOT gradients were higher in a standing than in a semi-supine position at rest implicating that standing might increase LVOTO [16].

This discrepancy may be explained by differences in patient characteristics and exercise modality. Patients in our study were older (mean age 61.5 ± 15.54 years) than those in *Joshi et al.* (mean age 56.4 ± 16 years) and may have been less physically resilient. Additionally, LVOT gradients provoked by treadmill ESE may be higher compared to bicycle ESE in a semi-supine position. This is supported by *Reant et al.*, who reports lower LVOT gradients during semi-supine bicycle ESE compared to treadmill ESE in 22 obstructive HCM patients [17]. This difference can possibly be primarily attributed to posture. During upright treadmill exercise and recovery, venous return is likely reduced compared to semi-supine bicycle ESE, resulting in lower preload, while systemic vasodilation decreases afterload and an elevated heartrate reduces filling times. Furthermore, body position also influences exercise physiology. A meta-analysis demonstrated that peak oxygen consumption (pVO₂) is higher in the upright than in the supine position, mainly due to greater muscle mass recruitment and higher peripheral oxygen extraction [18]. For HCM, this implies that while semi-supine ESE is advantageous for imaging, it may provoke lower pVO₂ values and consequently lower gradients compared with upright exercise modalities. The current AHA and ESC guidelines on obstructive HCM acknowledge the role of ESE but do not specify a preferred modality. This topic warrants future studies [1, 2].

### Squat exercise provokes the highest LVOT gradients

Squats are simple, rapid, cost-effective and require no additional equipment, making them an ideal bedside maneuver. The present findings are consistent with *Peng et al.* who evaluated maximal LVOT gradients in 109 HCM patients and reported higher gradients with squats compared to the Valsalva maneuver [19]. During squats, cardiac pre- and afterload increase due to augmented venous return flow and systemic vascular resistance. Rapid standing then causes a sudden decrease in pre- and afterload, thereby further augmenting LVOTO [20, 21]. *Dimitrow et al.* demonstrated that the simple change into the upright body position provoked an LVOT gradient in 8 of 37 patients [22] which underscores the influence of body posture. The addition of rapid exercise appears to be an effective form of exercise testing. In contrast, bicycle ESE, increases left ventricular stroke volume through adrenergic stimulation, while pre- and afterload are in general mildly reduced due to exercise-induced vasodilation, which in combination augments LVOTO [23]. The Valsalva maneuver primarily decreases venous return flow through elevated intrathoracic pressure leading to increased LVOTO [24].

The squat is a dynamic isotonic exercise containing an eccentric phase (lowing into the squat) and a concentric phase (rising back up) forming a dynamic resistance exercise. The repetitive nature of pre- and afterload changes during squats may partially explain their superior provocation of LVOTO in comparison to Valsalva maneuver or bicycle ESE. Additionally, the execution of squats can be readily standardized, thereby minimizing interobserver variability. In contrast, the performance of other bedside provocation maneuvers depends heavily on the examiner´s instructions, patient´s effort, or are difficult to standardize, as previously shown for the Valsalva maneuver [24]. Consequently, if the Valsalva maneuver fails to elicit a gradient of at least 50 mmHg in symptomatic patients with obstructive HCM, a squat maneuver, providing greater preload variation, should be considered as the next step before proceeding to ESE.

### Bicycle ESE is a considerable time burden in comparison to squats and the Valsalva maneuver

In healthcare systems, examination duration is an important consideration. To our knowledge, this study is the first to compare the duration of bicycle ESE with squats and the Valsalva maneuver in the context of LVOTO provocation in obstructive HCM. Bicycle ESE lasted on average 15 min and 54 s, significantly longer than squats with an average duration of 1 min and 23 s and the Valsalva maneuver with 15 s. Typical or recommended durations of bicycle ESE in the assessment of LVOTO in obstructive HCM have not been described previously. Guideline-recommended bicycle ESE protocols for ischemic heart disease suggest a procedural duration of up to 20 min, consistent with the findings of the present study [25]. Notably, additional time and personnel required for the setup and dismantling of the equipment necessary for bicycle ESE, as well as the time needed to change position for maneuver, such as squats, was not taken into consideration. Consequently, the actual durations of bicycle ESE and squats are likely underestimated. Previous studies by *Kumar et al.* and *Peng et al.* examined the effectiveness of the Valsalva maneuver in provoking LVOTO in obstructive HCM, with durations of > 10 s and 10–15 s which is in line with this study [26]. The squats in the present study lasted approximately 3 s per repetition, consistent with the protocol described by *Peng et al.* [19].

### Handgrip exercise does not significantly increase LVOT gradients in obstructive HCM

Handgrip exercise is increasingly used as a stress test in TTE, primarily in assessment of mitral valve regurgitation (MR) [7, 27, 28]. Previous studies demonstrated that isometric handgrip can increase the severity of mitral regurgitation in a substantial proportion of patients compared to resting conditions [7]. LVOTO is dynamic and dependent on pre- and afterload [16]. Isometric handgrip exercise increases afterload while preload remains largely unchanged [7]. Elevations of LVOT gradients are primarily driven by reduction in pre- and afterload, whereas handgrip exercise predominately increases afterload, thereby reducing left ventricular stroke volume and ejection velocity [29]. From a physiological perspective, a relevant increase in LVOT gradient was therefore not to be expected. Nevertheless, there are currently no studies investigating the influence of hand grip exercise on the LVOT gradient in obstructive HCM patients. The results of this study align with the physiological assumption that handgrip exercise does not significantly increase the maximum LVOT gradient. Consequently, it is concluded that handgrip exercise is not a suitable method for provoking the LVOT gradient in obstructive HCM.

### Limitations

This study has several limitations. The sample size was relatively small, and the investigation was conducted at a single tertiary care center, which may limit generalizability. In addition, the small cohort restricts statistical power, rendering the analyses primarily exploratory. The study population was heterogeneous with respect to age, NYHA class, and concomitant medication use, particularly beta-blockers, which may have influenced LVOT gradients. Most patients were referred for preliminary evaluation prior to potential myosin inhibitor therapy, explaining the low proportion of individuals with prior genetic testing or septal reduction therapy. Notably, no patient received myosin inhibitors at the time of examination. Additionally, the standardized fasting period implemented prior to TTE in this study to minimize the potential effect of recent food intake on LVOTO may not always be feasible in real-world clinical practice.

Bicycle ESE was used instead of treadmill ESE due to local equipment availability. Current guidelines on obstructive HCM do not specify a preferred ESE modality [1, 2]. However, semi-supine bicycle ESE may underestimate LVOTO gradients compared with upright treadmill protocols described in previous studies [17, 22, 30]. Another limitation is the fixed sequence of maneuvers, as fatigue or autonomic conditioning could have affected LVOT gradient measurements. Due to organizational constraints, the sequence could not be altered; however, sufficient recovery intervals and clinical monitoring were implemented to minimize potential measurement distortions. Additionally, it should be noted that the repetitive squat-to-exhaustion protocol used in this study differs from the simpler squat-to-stand maneuver described in current guidelines on HCM [1, 2]. Besides, as all participants had obstructive HCM, our study assessed gradient provocation rather than diagnostic specificity, which requires validation in non-obstructive HCM and control populations.

## Conclusion

The Valsalva maneuver, squats and bicycle ESE are all effective maneuvers in provoking LVOTO in obstructive HCM. Among these, squats produced the highest LVOT gradients in the majority of patients while requiring minimal time and resources, making them a practical adjunct to the Valsalva maneuver when results are inconclusive. However, their equivalence to bicycle ESE for diagnostic classification or treatment decision-making remains to be established. In contrast, isometric handgrip exercise did not significantly augment LVOT gradients and appears unsuitable as a provocation test in obstructive HCM.

## Supplementary Information

Below is the link to the electronic supplementary material.

Supplementary Material 1

## Data Availability

The datasets used and/or analysed during the current study are available from the corresponding author on reasonable request.
