# Supplementary material for: The effect of different exercise modalities on the maximal left ventricular outflow tract gradient in patients with obstructive hypertrophic cardiomyopathy
Source: Echo Res Pract. 2026 Oct 12;13:39. doi: 10.1186/s44156-026-00141-3 (PMC13656369; doi:10.1186/s44156-026-00141-3)
Supplement: Supplementary file 1 [file 44156_2026_141_MOESM1_ESM.pdf]

Supplemental Material – for online publication

Supplementary Figure 1

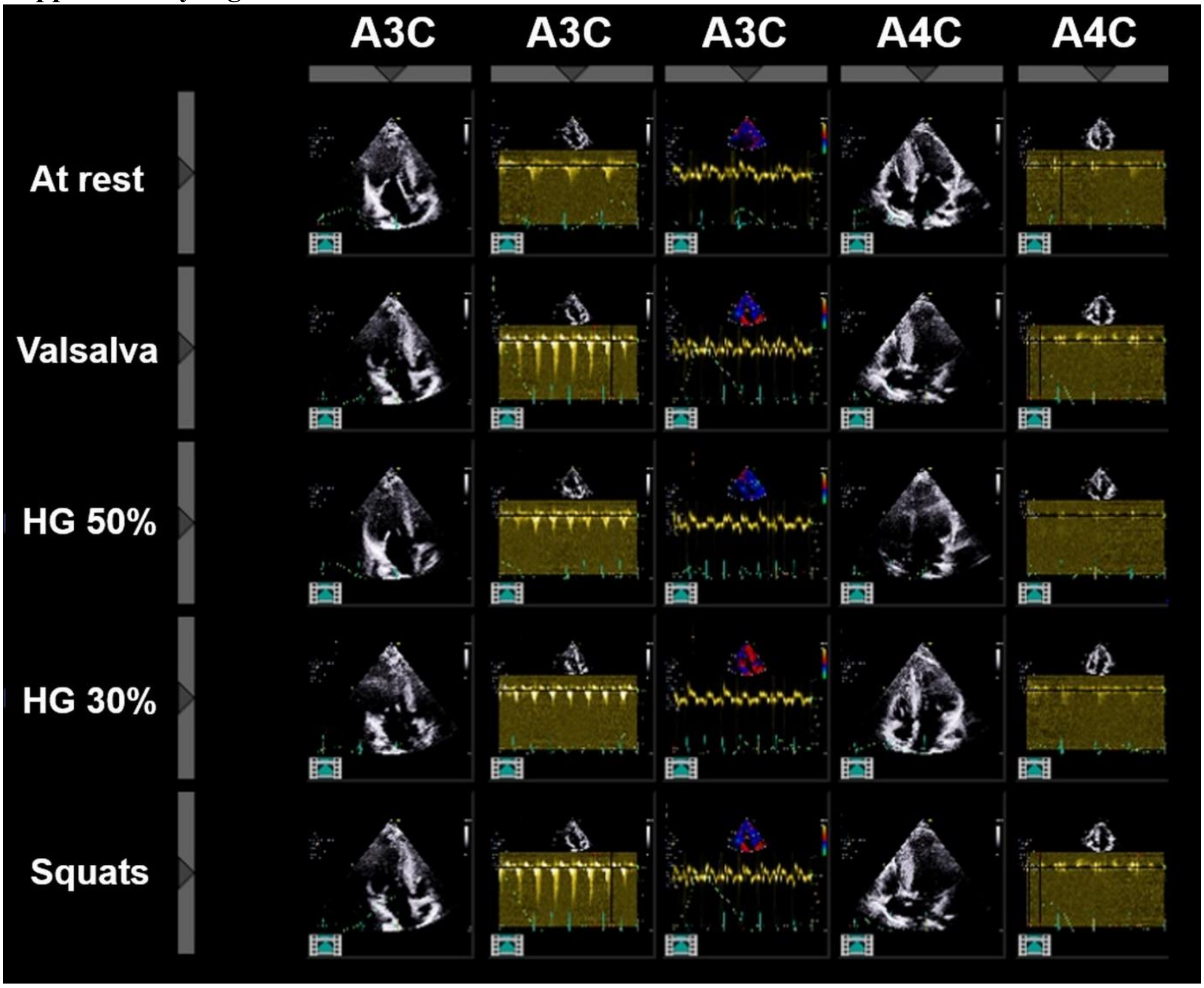

Supplementary Figure 1. Representative illustration of a standardized TTE protocol for LVOTO provocation. LVOTO was assessed first at rest, followed by the Valsalva maneuver, handgrip 50% of maximal force, handgrip 30% of maximal force and squats. CW-Doppler measurements of the maximal LVOT gradient were carried out in the A3C view. A3C: apical three-chamber view, A4C: apical four-chamber view, HG: handgrip.

Supplementary Figure 2

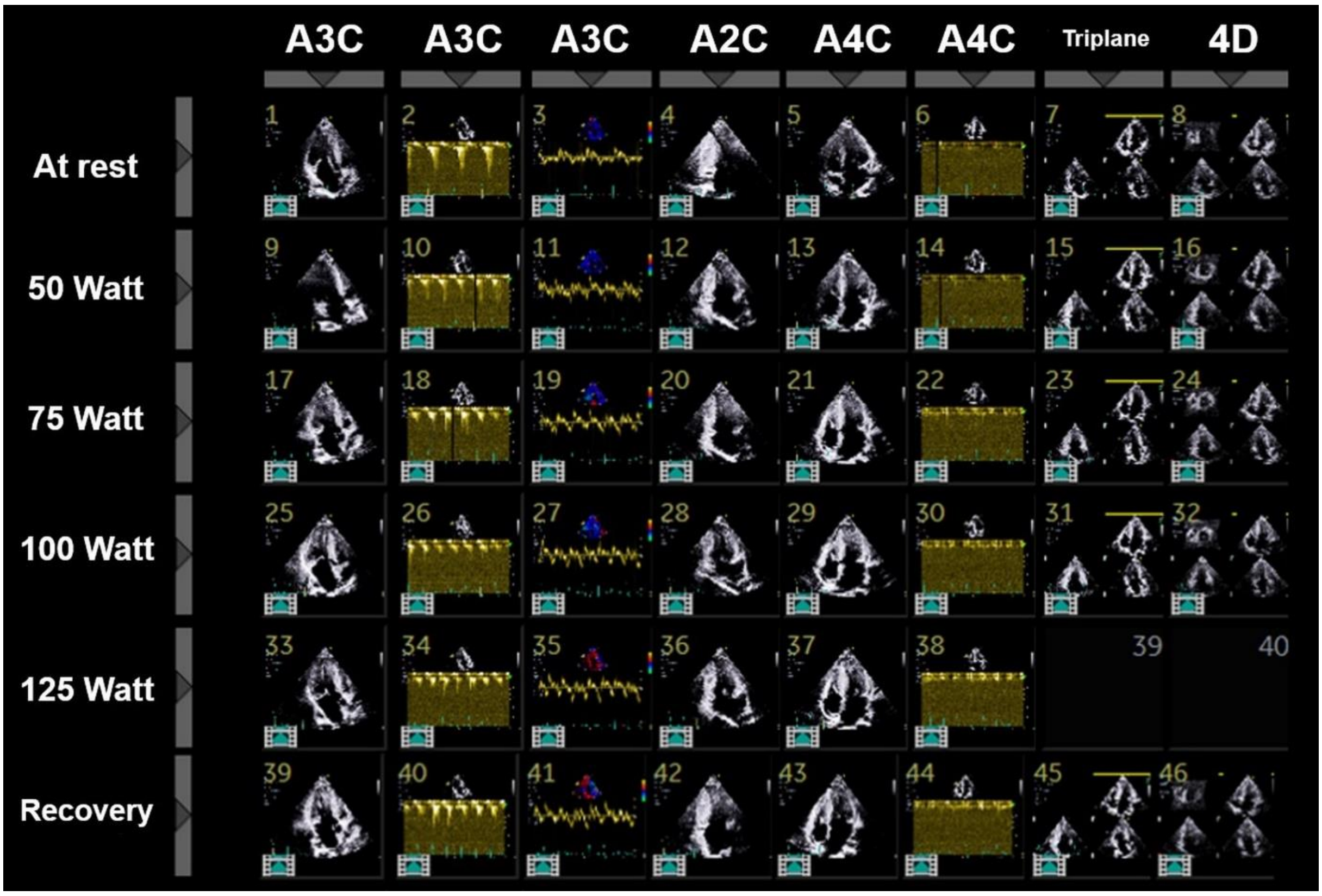

Supplementary Figure 2. Representative illustration of a standardized bicycle ESE protocol. The workload was increased in 25 Watt increments until the patient reached subjectively maximal exhaustion. LVOTO was assessed in the A3C view. A3C: apical three-chamber view, A2C: apical two-chamber view, A4C: apical four-chamber view, 4D: four-dimensional imaging.
